# Toll-Like Receptor Signaling Drives Btk-Mediated Autoimmune Disease

**DOI:** 10.3389/fimmu.2019.00095

**Published:** 2019-01-30

**Authors:** Jasper Rip, Marjolein J. W. de Bruijn, Marjolein K. Appelman, Simar Pal Singh, Rudi W. Hendriks, Odilia B. J. Corneth

**Affiliations:** ^1^Department of Pulmonary Medicine, Erasmus MC Rotterdam, Rotterdam, Netherlands; ^2^Department of Immunology, Erasmus MC Rotterdam, Rotterdam, Netherlands

**Keywords:** autoimmune disease, B cell, Bruton's tyrosine kinase, phosphoflow cytometry, Toll-like receptor

## Abstract

Bruton's tyrosine kinase (Btk) is a signaling molecule involved in development and activation of B cells through B-cell receptor (BCR) and Toll-like receptor (TLR) signaling. We have previously shown that transgenic mice that overexpress human Btk under the control of the CD19 promoter (CD19-hBtk) display spontaneous germinal center formation, increased cytokine production, anti-nuclear autoantibodies (ANAs), and systemic autoimsmune disease upon aging. As TLR and BCR signaling are both implicated in autoimmunity, we studied their impact on splenic B cells. Using phosphoflow cytometry, we observed that phosphorylation of ribosomal protein S6, a downstream Akt target, was increased in CD19-hBtk B cells following BCR stimulation or combined BCR/TLR stimulation, when compared with wild-type (WT) B cells. The CD19-hBtk transgene enhanced BCR-induced B cell survival and proliferation, but had an opposite effect following TLR9 or combined BCR/TLR9 stimulation. Although the expression of TLR9 was reduced in CD19-hBtk B cells compared to WT B cells, a synergistic effect of TLR9 and BCR stimulation on the induction of CD25 and CD80 was observed in CD19-hBtk B cells. In splenic follicular (Fol) and marginal zone (MZ) B cells from aging CD19-hBtk mice BCR signaling stimulated *in vitro* IL-10 production in synergy with TLR4 and particularly TLR9 stimulation, but not with TLR3 and TLR7. The enhanced capacity of CD19-hBtk Fol B cells to produce the pro-inflammatory cytokines IFNγ and IL-6 compared with WT B cells was however not further increased following *in vitro* BCR or TLR9 stimulation. Finally, we used crosses with mice deficient for the TLR-associated molecule myeloid differentiation primary response 88 (MyD88) to show that TLR signaling was crucial for spontaneous formation of germinal centers, increased IFNγ, and IL-6 production by B cells and anti-nuclear autoantibody induction in CD19-hBtk mice. Taken together, we conclude that high Btk expression does not only increase B cell survival following BCR stimulation, but also renders B cells more sensitive to TLR stimulation, resulting in increased expression of CD80, and IL-10 in activated B cells. Although BCR-TLR interplay is complex, our findings show that both signaling pathways are crucial for the development of pathology in a Btk-dependent model for systemic autoimmune disease.

## Introduction

B cells are crucial players in autoimmunity, as B cell depletion therapy was proven effective in patients with several systemic autoimmune diseases including Sjögren's syndrome (SjS) and rheumatoid arthritis (RA) (1, 2). These diseases are marked by altered B cell selection leading to the production of autoreactive antibodies, an important hallmark in the pathology of systemic autoimmune diseases.

Signaling via the B cell receptor (BCR) is essential for B cell survival (3). B cells are selected in the bone marrow (BM) at the large pre-B cell and the immature B cell stage for functional rearrangements of the immunoglobulin (Ig) heavy and light chain genes, respectively. Subsequently, checkpoints follow to select non-self-reactive B cells, both in the BM and in the periphery where maturing B cells, referred to as transitional B cells, undergo stringent selection. BCR signaling is crucial for the selection of B cells. However, evidence is accumulating that additional signals derived from CD40, Toll-like receptors (TLRs) and BAFFR also affect selection of B cells (4).

A crucial signaling molecule in the development, survival and activation of B cells is Bruton's tyrosine kinase (Btk), a member of the Tec family of non-receptor kinases. Btk is expressed in almost all cells of the hematopoietic lineage, except T cells and plasma cells (5, 6) and the expression of appropriate levels of Btk is crucial for normal B cell development (7, 8). Functionally, Btk is critically involved in many signaling pathways, such as BCR, TLR, and chemokine receptor signaling (9). Patients with loss-of-function mutations in the *BTK* gene present with X-Linked agammaglobulinemia (XLA), an inherited immunodeficiency marked by an almost complete arrest of B cell development at the pre-B cell stage in the BM and a near absence of peripheral B cells and circulating Ig (10, 11). In mice, Btk-deficiency does not result in an arrest in B cell development in the BM, although pre-B cell differentiation is somewhat impaired; due to a defective transitional B cell maturation the numbers of peripheral B cells are decreased (12–14). We have previously shown that BTK protein levels are different across human peripheral blood B cell subsets (15). Moreover, both in human and in mice BTK protein levels are upregulated when mature B cells are activated *in vitro* by various signals including those initiated by BCR, TLR, and CD40 stimulation (8). Taken together, these findings demonstrate the importance of Btk and indicate that its expression is tightly regulated.

We have generated transgenic mice that overexpress human Btk (hBtk) under the control of the CD19 promoter region (CD19-hBtk). B cells from these mice show increased survival and cytokine production and have the capacity to engage T cells in spontaneous germinal center (GC) formation (8). CD19-hBtk transgenic mice develop autoimmune pathology, characterized by lymphocyte infiltrates in several tissues including salivary glands and production of anti-nuclear autoantibodies (ANAs), which was observed from the age of 25 weeks onwards (8). This Btk-mediated autoimmunity phenotype is largely dependent on interaction with T cells (16) and resembles human systemic lupus erythematosus (SLE) and SjS. Human autoimmune disease is also associated with increased BTK expression: we recently showed that patients with RA and SjS have increased BTK protein levels in B cells from peripheral blood, compared with healthy controls (15). It remains unclear, however, whether the hBtk-mediated autoimmune phenotype in the mouse strictly depends on BCR signaling or on additional signaling pathways. The role of TLR signaling in the development of autoimmune diseases has been widely studied (17–25) and synergistic signaling of the BCR and TLRs has been implicated in systemic autoimmune disease in animal models (21, 26). Several lines of evidence indicate that Btk is critically involved in this BCR-TLR synergy. Btk can directly interact with the myeloid differentiation primary response 88 (MyD88) protein (27), an adaptor molecule downstream of many TLRs. Interestingly, TLR9 stimulation appears to affect B cell differentiation, as it was recently shown that engagement of TLR9, which recognizes dsDNA, can antagonize antigen processing and affinity maturation of antigen-specific B cells (28). The relevance of Btk in TLR-mediated B cell activation is supported by the finding that Btk-deficient B cells produced less IL-10 upon TLR9 stimulation compared with B cells with physiological Btk levels (29). In addition, Btk was shown to mediate synergistic signaling between the BCR and TLR9 (30), which is crucial for activation of autoreactive B cells (26). Therefore, it is conceivable that BCR-TLR synergy contributes to the initiation or maintenance of the autoimmune phenotype of BTK-overexpressing transgenic mice.

In this report, we aimed to determine the contribution of TLR signaling and BCR-TLR synergistic signaling to B cell activation in our mouse model of Btk-mediated autoimmune disease. Because the anti-dsDNA autoantibodies are prominent in aging CD19-hBtk transgenic mice (8), we focused hereby on TLR9. We first analyzed phosphorylation of various BCR and TLR downstream signaling molecules in splenic B cells. Consistent with increased survival of Btk-overexpressing B cells, when compared with wild-type (WT) B cells, CD19-hBtk transgenic B cells displayed increased phosphorylation of the ribosomal protein S6 upon BCR stimulation. Although the expression of TLR9 was reduced in CD19-hBtk B cells compared to WT B cells, a robust synergistic effect of TLR9 and BCR stimulation on S6 phosphorylation, CD25 and CD80 expression, and IL-10 production was observed in CD19-hBtk B cells. Together with the finding that TLR signaling was crucial for CD19-hBtk-mediated autoimmunity *in vivo*, these results point to a role of Btk in BCR-TLR synergy in the context of autoimmune disease development.

## Methods

### Mice and Genotyping

CD19-hBtk (31), Btk-deficient (12), and *IgH.TE*μ mice (32) were previously described. *Myd88*^LSL/LSL^ (*Myd88*^−/−^) mice (33) were crossed on CD19-hBtk mice to create the *Myd88*^−/−^CD19-hBtk line. Mice were genotyped by PCR and MyD88-sufficient non-hBtk transgenic littermates or C57/BL6 mice (Charles River) were used as WT controls. *Myd88*^−/−^ mice were crossed on *IgH.TE*μ mice (>F3 sv129xC57BL/6) and onset of leukemia was monitored every 3–6 weeks by peripheral blood screening for monoclonal B cell expansion. Mice were bred and kept under specified pathogen-free conditions in the Erasmus MC experimental animal facility. All experimental protocols were reviewed and approved by the Erasmus MC Committee of animal experiments (DEC).

### Flow Cytometry Procedures and Calcium Influx

Cell suspensions of spleen and BM were obtained using 100 μm cell strainers in magnetic-activated cell sorting (MACS) buffer (PBS/0.5% BSA/2mM EDTA), as previously described (16). 2 × 10^6^ cells were incubated with varying combinations of monoclonal antibodies (Table S1A) and stained according to previously described procedures, whereby isotype and fluorescence minus one (FMO) controls and non-expressing cells were used to set-up and validate the staining procedures (8). To stain for the isotype of intracellular immunoglobulins (Ig) in plasma cells, cells were fixed with BD Cytofix/Perm Buffer (BD Biosciences) and permeabilized with BD Perm/Wash Buffer (BD Biosciences). The eBioscience FoxP3 staining kit (eBioscience) was used to fix and permeabilize cells to stain for FoxP3 expression. For intracellular staining of cytokine-expressing cells, samples were fixed in PBS/2% paraformaldehyde and permeabilized and stained in MACS buffer containing 0.5% saponin (Sigma-Aldrich). For measuring intracellular calcium mobilization, 5 × 10^6^ splenocytes were incubated with fluorogenic probes Fluo3-AM and Fura Red-AM (Life Technologies), essentially as previously described (34), except that we stained for B220^+^ splenocytes. Cell cycle staining using propidium iodide (PI) was performed as previously described (14). Leukemic B cells (CD19^+^CD5^+^) were stained with FITC-labeled phosphatidylcholine (PtC) liposomes (DOPC/CHOL 55:45, Formumax Scientific Inc.) in MACS Buffer. All measurements were performed on an LSRII flow cytometer (BD Biosciences) and results were analyzed using FlowJo Version 9.7.6 software (TreeStar Inc).

### Phosphoflow Cytometry

To determine phosphorylation of intracellular proteins 0.5–1 × 10^6^ cells were cultured in 2% FCS in RPMI at 37°C for 5 min (pCD79a, pSyk and pPLCγ2) or for 3 h (pS6 and pAkt) with 20 μg/mL anti-mouse antigen-binding F(ab′)_2_ -IgM fragments (αIgM; Jackson ImmunoResearch), 2 μM (for CD79a, pPLCγ2 and pAkt) or 0.1 μM (pS6) CpG (ODN 1668; Invitrogen) or combinations thereof prior to fixation with the eBioscience FoxP3 staining kit Fix/Perm solution (eBioscience). Cells were centrifuged and washed twice with eBioscience FoxP3 staining kit Perm/Wash solution (eBioscience) and subsequently stained for 30 min at 4°C with markers to identify B cells and T cells (Table S1B), followed by staining for the appropriate phospho-target (30 min at RT). Cells stained for pS6 were subsequently stained with anti-rabbit PE antibody (Jackson ImmunoResearch) 15 min at RT. Isotype and FMO controls were included in the set-up of the staining procedure, verifying the signal intensities of the phospho-targets. In addition, non-expressing T cells were used as internal controls for BCR-restricted signaling molecules in all experiments. All measurements were performed on an LSRII flow cytometer (BD Biosciences), and results were analyzed using FlowJo Version 9.7.6 software (TreeStar Inc).

### *In vitro* Stimulation for Cytokine Expression

To measure cytokine-expressing lymphocytes, splenic cell suspensions were stimulated for 4 h at 37°C using 50 ng/ml Phorbol 12-myristate 13-acetate (PMA) and 500 ng/ml ionomycin (both Sigma-Aldrich), 10 μg/mL αIgM (Jackson ImmunoResearch), 200 μg/mL Poly:IC (Invivogen), 1.6 μg/mL Lipopolysaccharide (LPS; Sigma-Aldrich), 40 μg/mL imidazoquinoline (Imiquimod VacciGrade™; Invivogen), and/or 2 μM CpG (ODN 1668; Invitrogen) in combination with monensin (Golgistop; BD Biosciences). To evaluate the effect of Btk inhibition, 1 μM ibrutinib (PCI-32765; Sigma-Aldrich) was added to the *in vitro* cultures.

### MACS Purification and *in vitro* B Cell Cultures

Splenic cell suspensions from CD19-hBtk and WT control mice were prepared in MACS buffer. MACS procedure and culture was performed as previously published (8). Cells were stained with biotinylated antibodies (Table S1C) followed by streptavidin-coupled magnetic beads (Miltenyi Biotec) and unlabeled naïve B2 cell fractions were collected by magnetic depletion of labeled cells with a purity of >92%. Purified naïve B cells were stimulated for 48 h to evaluate cell cycle progression and 48 or 72 h for activation markers with 10 μg/mL αIgM (Jackson ImmunoResearch), 2 μM CpG (ODN 1668; Invitrogen) or combinations thereof in culture medium (RPMI 1640/ 10% FCS/ 50 μg/mL gentamycin/ 0.05 mM ß-mercaptoethanol). To evaluate the effect of Btk inhibition, 1 μM ibrutinib (PCI-32765) was added to *in vitro* culture conditions.

### Immunohistochemistry

*Myd88*^−/−^CD19-hBtk, CD19-hBtk, and age-matched WT control mice were sacrificed at 28–33 weeks of age. Salivary glands and kidneys were collected, embedded in O.C.T-compound (Sakura) and stored at −80°C. Immunohistochemical staining was performed as previously described (35). Slides were washed with PBS and stained for 60 min with anti-CD3 (eBioscience), anti-IgM or anti-IgG2c rat anti-mouse antibodies (BD Biosciences), followed by a counterstaining with anti-rat Alkaline Phosphatase (AP)-labeled antibodies (Jackson ImmunoResearch). Anti-CD3-stained slides were stained for 60 min with IgM^FITC^ rat anti-mouse antibodies (BD Biosciences), followed by counterstaining with streptavidin, anti-FITC or anti-PE Peroxidase (PO)-labeled antibodies (Rockland). Slides were embedded in Kaiser glycerol gelatin (Merck).

### HEp-2 Reactivity Assay

Serum samples of 28–33 week-old mice (diluted 1:100 in PBS) were incubated on HEp-2 slides (Bio-Rad Laboratories) for 60 min, as previously described (8). After washing with PBS, slides were incubated with Alexa Fluor-488 conjugated donkey anti-mouse IgM or IgG F(ab′)_2_ fragments (Jackson ImmunoResearch) for 1 h. After washing and staining for 5 min with DAPI, slides were embedded in VectaShield (Vector Laboratories). The LSM 510 META confocal fluorescence microscope (Zeiss) was used to measure fluorescence intensity.

### ELISA

Serum Ig subclasses were determined by sandwich ELISA. First, plates were coated with unlabeled anti-IgM, anti-IgG1, and anti-IgG2a (Southern Biotech) overnight at 4°C. The next day, serum and isotype standards (IgM, Bio-Rad; IgG1 and IgG2a, Southern Biotech) were serially diluted and incubated at RT for 3 h. This was followed by an incubation of 30 min with biotinylated IgM, IgG1, and IgG2a-specific antibodies (Southern Biotech) and subsequently 30 min of incubation with streptavidin peroxidase-labeled antibodies (Jackson ImmunoResearch). Finally, we added 3, 3',5, 5'- Tetramethylbenzidine (TMB) substrate (SeraCare) and stopped the reaction by using sulfuric acid. Samples were read at an OD of 450 nm using the VersaMax Microplate Reader (Molecular Devices) to measure color intensity.

### Line Immunoblot Assay (LIA) for Extractable Nuclear Antigens

To measure extractable nuclear antigens, we used the INNO-LIA® ANA Update kit (Fujirebio) according to manufacturer's instructions. In short, LIA strips were incubated with serum samples of 28–33 week-old mice [diluted 1:200 in sample diluent (Fujirebio)] or with cut-off control containing human IgG positive control antibodies. Next, LIA strips were incubated with AP-labeled anti-mouse IgG antibodies (Jackson ImmunoResearch). The cut-off control was incubated with the supplied AP-labeled anti-human IgG antibodies (Fujirebio). Finally, we added 5-bromo-4-chloro-3-indolyl phosphate (BCIP)/nitro blue tetrazolium (NBT) substrate (Fujirebio) diluted in substrate buffer and stopped the reaction by adding sulfuric acid (Fujirebio). The LIA strips were removed from the troughs and interpreted after they had completely dried. As reference, LIA strips from samples were compared to the supplied cut-off control and regarded positive when bands were stained more intensely than the cut-off control.

### Statistical Analysis

The non-parametric Mann-Whitney *U* test was used for statistical analyses. Log Rank test was used to calculate the significance for survival differences between indicated group of mice for the chronic lymphocytic leukemia (CLL) experiments. Differences between groups with *P*-values below 0.05 were considered significant. Statistical analysis was performed using GraphPad Prism 5 software (GraphPad Software Inc).

## Results

### CD19-hBtk Transgenic B Cells Display Increased Signaling of the Akt Pathway

**Figure 1:**
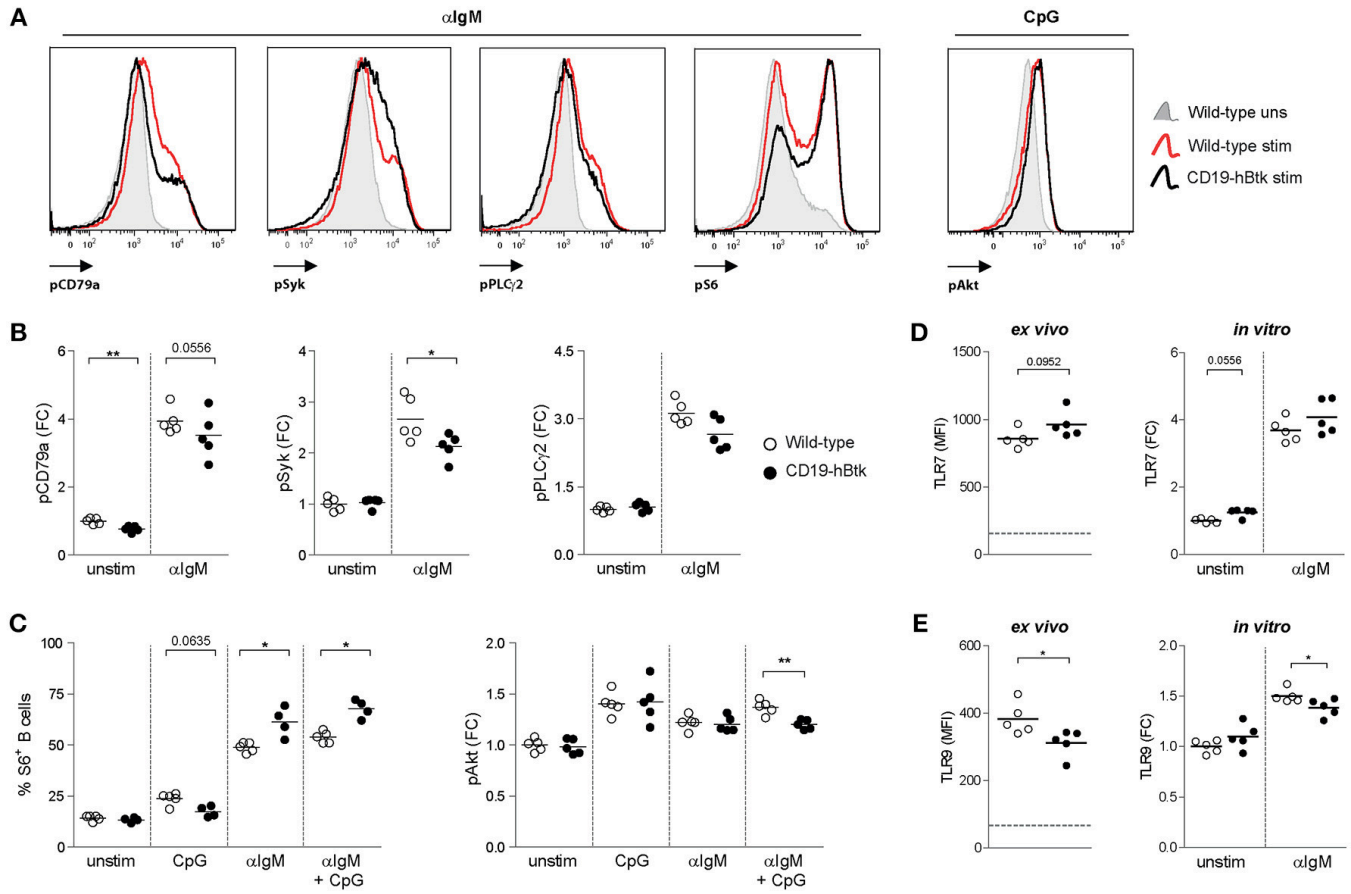
Increased S6 phosphorylation upon BCR engagement in CD19-hBtk B cells. **(A)** Histogram overlays of representative examples of the phosphoprotein analyses are shown for αIgM-induced induction of pCD79a, pSyk, pPLCγ2 and pS6, as well as CpG-induced pAkt. **(B–C)** Splenic cells of wild-type (WT) and CD19-hBtk transgenic mice were stimulated for 5 min (CD79a, Syk, and PLCγ2) or 3 h (S6 and Akt) and gated for B cells after the indicated *in vitro* stimulation. Fold change (FC) increase of median fluoresence intensity (MFI) values compared to WT unstimulated are shown for phosphorylation of CD79a, Syk, PLCγ2 **(B)**, and Akt **(C)**. Phosphorylation of ribosomal protein S6 was quantified using percentages of positive cells compared to unstimulated cells **(C)**. **(D,E)** MFI values of TLR7 **(D)** and TLR9 **(E)** protein in splenic B cells *ex vivo* and MFI FC induction of these TLRs on MACS-purified B cells after 48 h of stimulation with αIgM (dotted line indicates expression in T cells). Symbols represent individual mice and bars indicate mean values. Graphs represent one to two individual experiments, each with 4–5 mice per group; CD19-hBtk and WT mice were 8–10 weeks old; ^*^p < 0.05, ^**^p < 0.01 by Mann-Whitney *U* test.

Given the increased survival and activated phenotype of Btk-overexpressing (CD19-hBtk) B cells (8, 15), we first studied BCR signaling in splenic B cells from 8-week-old CD19-hBtk mice. To this end, we stimulated total WT and CD19-hBtk splenic cells with αIgM, the TLR9 ligand CpG, or combinations thereof and determined the phosphorylation status of various signaling molecules by phosphoflow cytometry analysis of gated B220^+^CD3^−^ B cells (Figure 1A; gating strategy in Figure S1A). In these experiments, we used unstimulated gated B220^+^CD3^−^ B cells as a control.

Phosphorylation of Y182 in the ITAM of Ig-α/CD79a, the transmembrane protein that forms a complex with the BCR, was reduced in unstimulated CD19-hBtk B cells compared with WT controls (Figure 1B). Expression of phosphorylated Y759 PLCγ2 (pPLCγ2) and Y348 Syk (pSyk) was similar in unstimulated WT and CD19-hBtk B cells (Figure 1B). As expected, in WT B cells phosphorylated CD79a (pCD79a) and pPLCγ2 were induced upon αIgM stimulation (Figures 1A,B), but not upon CpG stimulation for CD79a and pPLCγ2 (Figure S1B). Both pCD79a and pPLCγ2 appeared somewhat reduced in αIgM-stimulated CD19-hBtk B cells, although this was not significant. BCR-induced pSyk was significantly reduced in CD19-hBtk B cells when compared with WT B cells (Figure 1B).

TLR9-induced phosphorylation (S240/S244) of S6, a ribosomal protein that is a downstream target of various signaling cascades, including the Akt pathway, appeared unchanged in CD19-hBtk B cells compared with WT controls (Figure 1C). However, significantly increased pS6 was seen in CD19-hBtk B cells upon BCR engagement alone or in combination with CpG stimulation (Figure 1C). Phosphorylation of Akt at S473/T308 upon stimulation with αIgM or CpG was comparable between CD19-hBtk and WT B cells, although CD19-hBtk B cells showed reduced pAkt upon combined BCR-TLR stimulation (Figure 1C). Taken together, these findings revealed limited effects of TLR stimulation on the BCR signaling pathway, whereas BCR stimulation induced S6 phosphorylation more strongly in CD19-hBtk than in WT B cells.

In addition, we investigated the expression levels of TLR7 and TLR9 protein as these TLRs are most relevant in the context of autoimmune disease (17–19, 23). Expression of TLR7 was similar in CD19-hBtk and WT splenic B cells *ex vivo* and following *in vitro* αIgM stimulation (Figure 1D). In contrast, TLR9 protein levels were decreased in CD19-hBtk B cells compared with WT controls, both *ex vivo* and upon 48 h of *in vitro* stimulation with αIgM (Figure 1E). This finding implies that CD19-hBtk and WT may have a different responsiveness to TLR9 ligands.

### BCR and TLR9 Signaling Differentially Affect CD19-hBtk B Cell Proliferation and Survival

**Figure 2:**
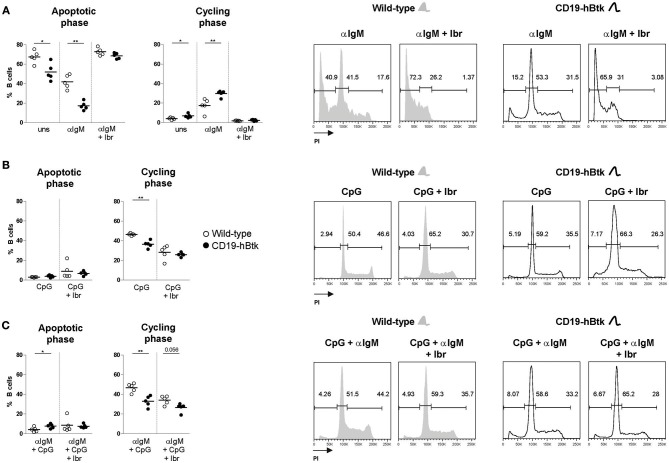
BCR and TLR9 stimulation have different effects on CD19-hBtk B cells. Proportions of B cells in apoptotic or cycling fractions, as determined by propidium iodide (PI) DNA content staining of *in vitro* cultured purified naïve splenic B cells that were stimulated with 10 μg αIgM **(A)**, 2 μM CpG **(B)**, or αIgM and CpG combined **(C)**, with or without ibrutinib (Ibr, 1 μM) as indicated (left). Symbols represent individual mice and bars indicate mean values. Representative flow cytometry graphs of WT and CD19-hBtk B cells are shown on the right, with the gating applied for apoptotic cells (sub-G1), resting cells (G0/G1), and dividing (S/G2/M) B cells. CD19-hBtk and WT mice were 8–10 weeks old; *n* = 4–5 per group; ^*^p < 0.05, ^**^p < 0.01 by Mann–Whitney *U*-test.

To study whether TLR9 responsiveness alters BCR-mediated activation of CD19-hBtk B cells, we MACS-purified naïve B cells from spleens of 8-week-old CD19-hBtk and WT mice and stimulated these fractions *in vitro* with αIgM, CpG, or a combination thereof for 48 h to evaluate cell cycle progression by PI staining. Consistent with our published findings (8), these analyses showed that upon BCR stimulation survival and proliferation was increased in B cells from CD19-hBtk mice, compared with WT littermates (Figure 2A). This effect was entirely dependent on Btk kinase activity, as the presence of the Btk small molecule inhibitor ibrutinib completely abrogated cellular proliferation in both WT and CD19-hBtk αIgM stimulated B cells (Figure 2A). In contrast, in single CpG-stimulation and combined αIgM/CpG-stimulation, Btk-overexpressing B cells showed reduced proliferation, compared with WT B cells (Figures 2B,C). This was dependent on Btk kinase activity, as Btk inhibition essentially leveled-out the differences between CD19-hBtk and WT B cells (Figures 2B,C). Intriguingly, addition of ibrutinib decreased the proliferation of CpG-stimulated WT B cells, suggesting that Btk kinase is part of the signaling pathway downstream of TLR9 that induces B cell proliferation. Therefore, both Btk inhibition and Btk overexpression resulted in reduced B cell proliferation following stimulation by CpG.

In summary, we found that Btk overexpression increases survival and proliferative responses upon BCR engagement, but limits the responsiveness to TLR9 stimulation.

### The Activation Status of CD19-hBtk B Cells Is Increased Following Combined BCR and TLR9 Stimulation

**Figure 3:**
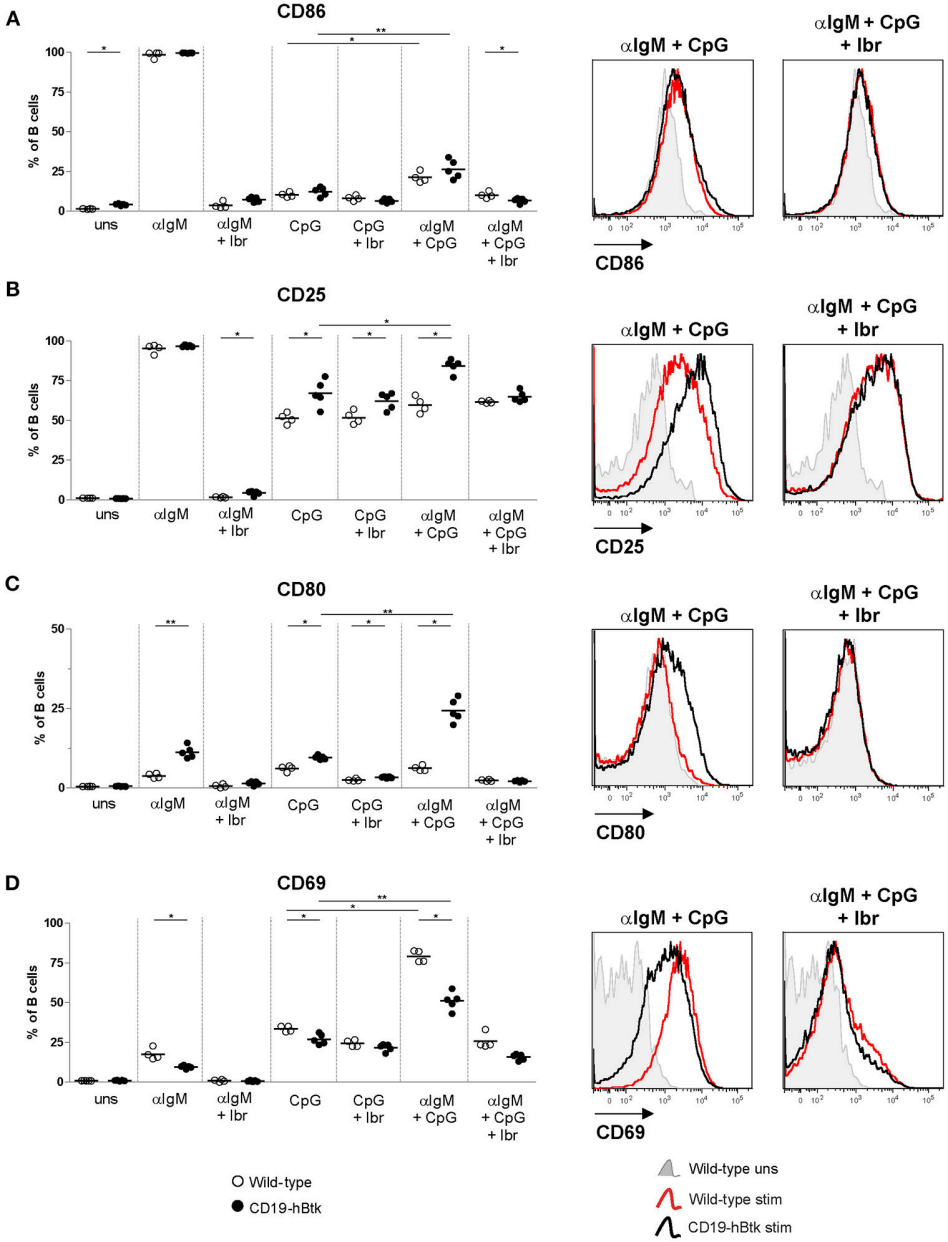
CD19-hBtk B cells show increased upregulation of activation markers upon synergistic BCR and TLR9 stimulation. Proportions of B cells positive for CD86 **(A)**, CD25 **(B)**, CD80 **(C)**, and CD69 **(D)** upon 72 h of stimulation of MACS-purified naïve splenic B cells with 10 μg αIgM (αIgM) and/or 2 μM CpG, with or without ibrutinib (Ibr, 1 μM), as indicated (left). Symbols represent individual mice and bars indicate mean values. Representative histogram overlays are depicted on the right. CD19-hBtk and WT mice were 8–10 weeks old; *n* = 4–5 per group; ^*^p < 0.05, ^**^p < 0.01 by Mann–Whitney *U*-test.

Next, we tested activation marker upregulation of naïve B cells of 8-week-old mice upon *in vitro* stimulation with αIgM, CpG, and a combination thereof for 72 h. No major differences were observed between WT vs. CD19-hBtk B cells in the upregulation of CD86 expression (Figure 3A). *In vitro* stimulation with CpG or αIgM resulted in an induction of CD25/IL-2R and the costimulatory protein CD80 in B cells from both mouse groups (Figures 3B,C). Combined stimulation of αIgM and CpG decreased surface expression levels of CD86 and CD25 compared to αIgM alone, both in WT and in CD19-hBtk B cells (Figures 3A,B), whereas this was not the case for CD80 (Figure 3C). However, following CpG stimulation surface expression of CD25 and CD80 was significantly increased on CD19-hBtk B cells compared with WT B cells (Figures 3B,C). Interestingly, in CD19-hBTK B cells but not in WT B cells, simultaneous stimulation with CpG and αIgM resulted in an additional increase in CD25 and particularly CD80 expression compared to CpG alone (Figure 3C). Expression of the early activation marker CD69 was lower in CD19-hBtk B cells, compared with WT B cells upon stimulation with αIgM, CpG, or combined stimulation (Figure 3D). As early activation marker CD69 is downregulated shortly after its induction, it is conceivable that its reduced expression on CD19-hBtk B cells resulted from rapid downregulation. Our finding of reduced CD69 expression would thus be consistent with enhanced activation of CD19-hBtk B cells following BCR or TLR9 stimulation. In these experiments, Btk inhibition by ibrutinib affected BCR-induced but not TLR9-induced changes in the expression of surface activation markers (Figures 3A–D).

Therefore, we conclude that BCR and TLR9 signaling act in synergy to induce a more activated surface phenotype in Btk-overexpressing B cells.

### IL-10-Production by CD19-hBtk B Cells Is Synergistically Increased Following BCR and TLR Stimulation

**Figure 4:**
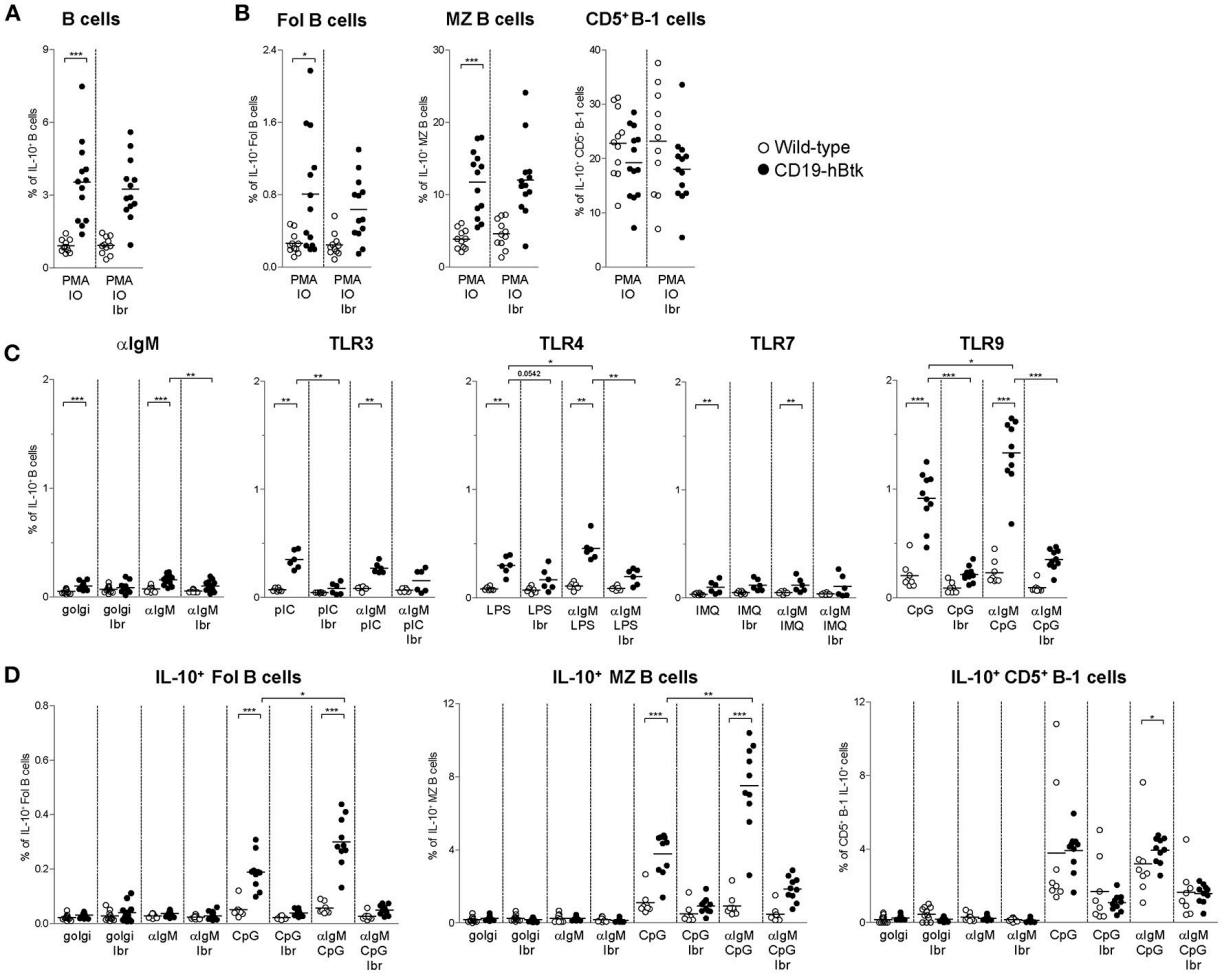
Increased IL-10 expression following synergistic BCR and TLR9 stimulation of CD19-hBtk B cells. **(A,B)** Proportions of IL-10-expressing B cells after 4 h of *in vitro* stimulation of total splenocytes from the indicated mice with PMA/ionomycin in the presence of monensin (golgi), as determined by intracellular flow cytometry. Shown are data for gated total CD19^+^B220^+^CD3^−^ B cells **(A)**, or for gated B cell subpopulations **(B)**, as indicated: follicular (Fol) B cells, marginal zone (MZ) B cells and CD5^+^ B-1 cells. **(C)** Proportions of IL-10-expressing B cells after 4 h of *in vitro* stimulation of total splenocytes from the indicated mice with αIgM, the indicated TLR ligands, or combinations thereof in the presence of monensin (golgi), as determined by intracellular flow cytometry. **(D)** Proportions of IL-10-expressing B cells after 4 h of *in vitro* stimulation of total splenocytes from the indicated mice with αIgM (αIgM), CpG, or combinations thereof, with or without ibrutinib (Ibr, 1 μM), in the presence of monensin (golgi), as determined by intracellular flow cytometry. CD19-hBtk and WT mice were 28–33 weeks old. Symbols represent individual mice and bars indicate mean values. Graphs represent two to three individual experiments; ^*^p < 0.05, ^**^p < 0.01, ^***^p < 0.001 by Mann–Whitney *U*-test.

We have previously shown that autoimmune CD19-hBtk B cells of 30-week-old (aged) mice have an enhanced capacity to produce various cytokines, including IL-6, IL-10, and IFNγ (16). Accordingly, stimulation with PMA/ionomycin increased the proportions of IL-10-producing CD19-hBtk transgenic B cells to higher levels than WT control B cells (Figure 4A). This increase was observed in follicular (Fol) and marginal zone (MZ) B cells, but not in splenic CD5^+^ B-1 cells, and was not affected by the presence of ibrutinib (Figure 4B; gating strategy in Figures S2A,B). To study B cell responsiveness to BCR and TLR signaling with respect to IL-10 production, we stimulated splenocytes from 30-week-old WT and CD19-hBtk mice with αIgM, poly-IC (pIC; recognized by TLR3), LPS (recognized by TLR4), Imiquimod (IMQ; TLR7), and CpG (TLR9), either alone or in various combinations in the presence of golgistop (monensin). In splenocyte cultures with monensin alone or with αIgM very few B cells were positive for IL-10, as measured by intracellular flow cytometry (Figure 4C). TLR stimulation with LPS and particularly CpG resulted in a clear induction of IL-10^+^ B cells specifically in CD19-hBtk mice (Figure 4C). The proportions of IL-10^+^ CD19-hBtk B cells—but not WT B cells—synergistically increased upon combined stimulation of BCR/TLR4 and BCR/TLR9 (Figure 4C). Stimulation with αIgM, together with TLR3 or TLR7 did not show a synergistic effect. This capacity of transgenic Btk was largely dependent on its kinase activity, because ibrutinib reduced IL-10 production in response to TLR and BCR ligands nearly to WT levels (Figure 4C).

Aged CD19-hBtk mice have increased numbers of splenic CD5^+^ B-1 cells (8) and slightly decreased numbers of Fol and MZ B cells, suggesting differential effects of Btk overexpression on these B cell subpopulations. Therefore, we investigated IL-10 production by splenic B cell subsets separately. The proportions of IL-10^+^ Fol B cells were low (Figure 4D). Nevertheless, we noticed that IL-10^+^ Fol B cells were increased in CD19-hBtk mice upon stimulation with CpG. Hereby, a synergistic effect was observed when B cells were additionally stimulated with αIgM (Figure 4D). MZ B cells of CD19-hBtk mice contained the highest proportions of IL-10^+^ cells upon synergistic BCR and TLR9 stimulation (Figure 4D). We observed that splenic CD5^+^ B-1 cells from CD19-hBtk mice did not show substantial differences, although significant, in proportions of IL-10^+^ cells, when compared with CD5^+^ B-1 cells from WT littermates (Figure 4D).

These data show that, compared to WT B cells, IL-10-production is increased following synergistic BCR and TLR9 stimulation in CD19-hBtk Fol and MZ B cells, but not in CD5^+^ B-1 B cells. Hereby, CD19-hBtk transgenic MZ B cells have the highest capacity to produce IL-10.

### Increased IFNγ and IL-6 Production by CD19-hBtk B Cells Reflects *in vivo* B Cell Activation

**Figure 5:**
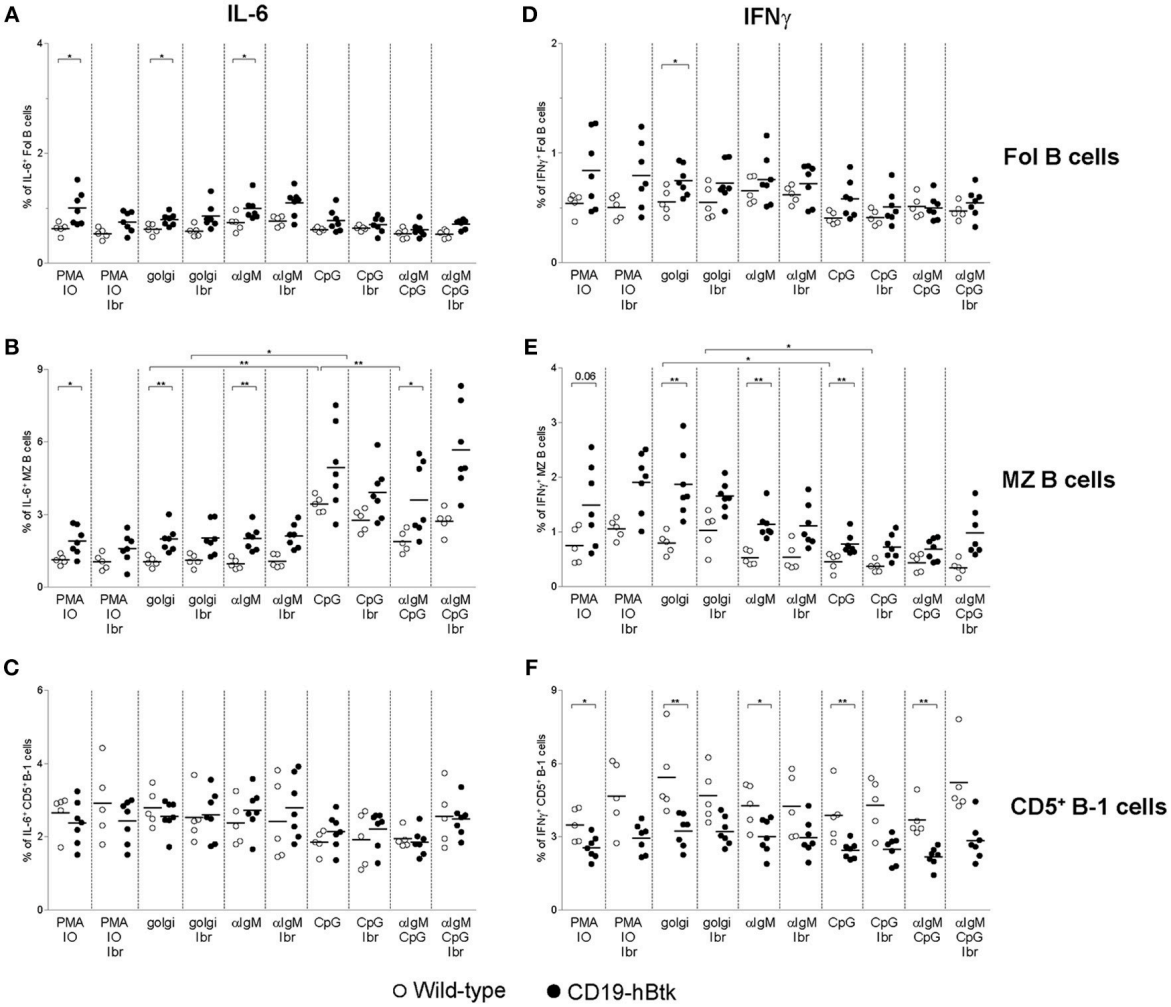
Analysis of IL-6 and IFNγ expression in B cell subsets from CD19-hBtk B cells. Proportions of IL-6^+^ **(A–C)** and IFNγ^+^ **(D–F)** B cells upon stimulation with the indicated stimuli, with or without ibrutinib (Ibr, 1 μM) in the presence of monensin (golgi), within gated B cell subsets: follicular (Fol) B cells **(A,D)**, MZ B cells **(B,E)**, and CD5^+^ B-1 cells **(C,F)**. CD19-hBtk and WT mice were 28–33 weeks old. Symbols represent individual mice and bars indicate mean values. Graphs are representative for one to two individual experiments; ^*^p < 0.05, ^**^p < 0.01 by Mann–Whitney *U*-test.

Splenic B cells from aged CD19-hBtk mice have increased proportions of IL-6^+^ and IFNγ^+^ cells upon stimulation with PMA/ionomycin, compared to those from WT mice (16). A separate analysis of splenic B cell subsets showed that the increased IL-6^+^ and IFNγ^+^ production was present in CD19-hBtk transgenic Fol and MZ B cells, but not in CD19-hBtk transgenic CD5^+^ B-1 cells (Figure 5; gating strategy in Figures S2A,B). Hereby, the proportions of IL-6^+^ or IFNγ^+^ cells were larger in the MZ B cell than in the Fol B cell fractions. We found that both for WT and for transgenic mice the proportions of IL-6^+^ and IFNγ^+^ cells were quite similar in cultures with monensin alone and in cultures stimulated by PMA/ionomycin or αIgM, irrespective of the presence of ibrutinib (Figure 5). Thus, addition of ibrutinib to these *in vitro* cultures did not reduce the proportions of IL-6^+^ or IFNγ^+^ CD19-hBtk Fol or MZ B cells to WT levels (Figures 5A,B,D,E).

*In vitro* CpG stimulation, either alone or in combination with αIgM or ibrutinib had limited effects on IL-6^+^ or IFNγ^+^ production by Fol B cell or CD5^+^ B-1 cells (Figures 5A,C,D,F). In contrast, CpG stimulation increased the proportions of IL-6^+^ cells by MZ B cells from both CD19-hBtk as WT mice, to levels higher than in PMA/ionomycin cultures (Figure 5B). Addition of ibrutinib *in vitro* did not appear to affect the production of IL-6 by MZ B cells from either mouse group; additional stimulation with αIgM reduced the frequencies of IL-6^+^ cells, compared to CpG stimulation alone (Figure 5B). In contrast to IL-6 production, we noticed that CpG stimulation was associated with moderately reduced IFNγ production by MZ B cells, irrespective of the presence or absence of ibrutinib (Figure 5E).

In summary, the increase of the proportions of IFNγ^+^ and IL-6^+^ cells within the B cell population present in the spleen of CD19-hBtk mice could be attributed to Fol and MZ B cells and not to CD5^+^ B-1 cells. This increase was largely independent of *in vitro* stimulation and thus essentially reflected *in vivo* B cell activation. Only IL-6 production by MZ B cells could be enhanced *in vitro* by CpG stimulation.

### Increased BCR Responsiveness in CD19-hBtk B Cells Is Independent of MyD88 Expression

**Figure 6:**
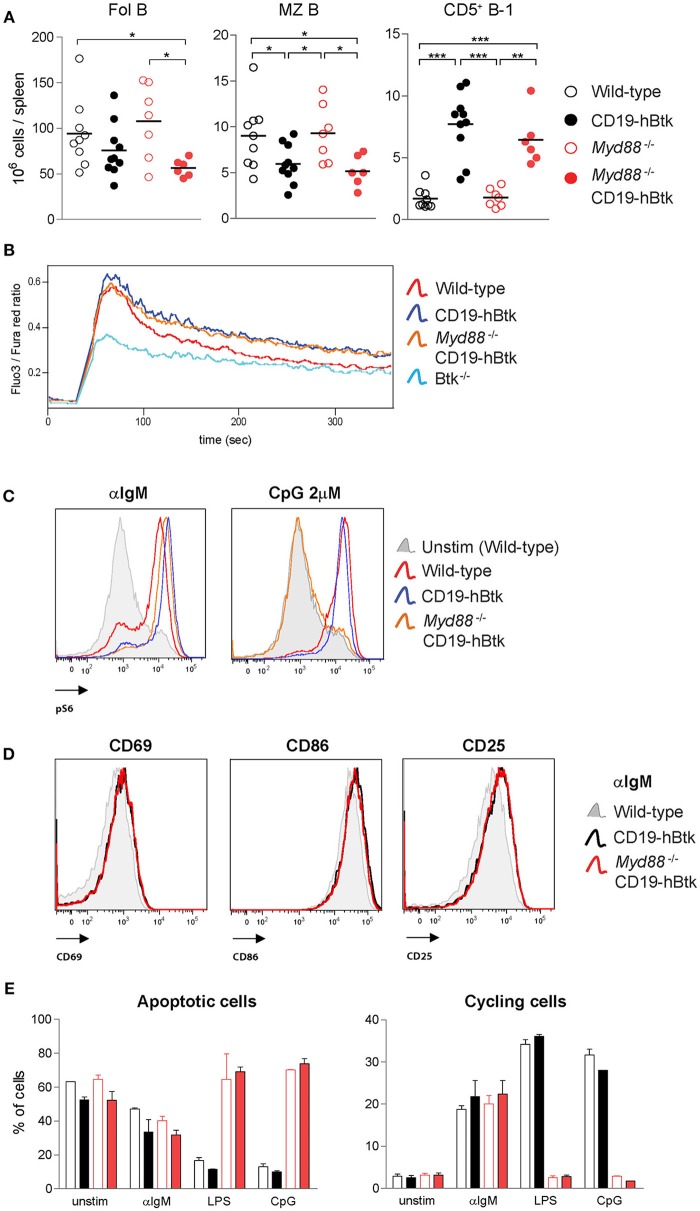
Increased BCR responsiveness in CD19-hBtk B cells is independent of MyD88 expression. **(A)** Quantification of the absolute numbers of Fol B cells (CD19^+^CD21^−^CD23^+^), MZ B cells (CD19^+^CD21^+^CD23^−^), and CD5^+^ B-1 cells (CD19^high^B220^int^CD5^+^) in spleens from the indicated aged mice. **(B)** Ca^2+^ influx assay in B cells after stimulation with 25 μg F(ab')_2_ anti-IgM in the indicated mouse groups. Data are representative for three mice analyzed. **(C)** Representative histogram overlays of phosphorylated S6 (pS6) upon 20 μg/mL αIgM or 2 μM CpG stimulation in the indicated mouse groups. Data are representative for two to three mice analyzed. **(D)** Representative histogram overlays of the expression of activation markers CD69, CD86, and CD25 in *Myd88*^−/−^ CD19-hBtk (red), CD19-hBtk (black), and WT mice (gray). Data are representative for two mice analyzed. **(E)** Proportions of B cells in apoptotic (left) or cycling (right) fractions, as determined by PI staining for DNA content, after 2 days of *in vitro* stimulation with the indicated stimuli. CD19-hBtk and WT mice were 28–33 weeks old **(A)** or eight weeks old **(B–E)**. Data (mean values + SD) represent one to three individual experiments; ^*^p < 0.05, ^**^p < 0.01, ^***^p < 0.001 by Mann–Whitney *U*-test.

To study whether TLR signaling is important for the development of Btk-mediated autoimmune disease, we crossed CD19-hBtk mice onto a MyD88-deficient background and aged these mice to characterize their phenotype. At ~30 weeks of age, the total numbers of Fol and MZ B cells in the spleen were decreased in both CD19-hBtk and *Myd88*^−/−^CD19-hBtk mice, compared to WT and *Myd88*^−/−^ mice (Figure 6A). The absolute numbers of CD5^+^ B-1 cells showed the converse and were increased in CD19-hBtk and *Myd88*^−/−^ CD19-hBtk mice (Figure 6A).

When we investigated BCR responsiveness of total splenic B cells of 8-week-old mice, we found that the prolonged calcium influx and increased S6 phosphorylation upon αIgM stimulation of CD19-hBtk B cells was MyD88-independent (Figures 6B,C). *Myd88*^−/−^ CD19-hBtk B cells did not increase pS6 levels upon CpG stimulation (Figure 6C), confirming that these cells were TLR unresponsive. In addition, we noticed that the enhanced upregulation of the cell surface markers CD69, CD86, and CD25 upon αIgM stimulation was similar for CD19-hBtk and *Myd88*^−/−^ CD19-hBtk B cells (shown for 48 h; Figure 6D). Cell cycle analysis by PI staining after 2 days of *in vitro* culture of MACS-purified B cell fractions, in the presence or absence of αIgM, revealed increased survival and proliferation of CD19-hBtk B cells in a MyD88-independent manner (Figure 6E). As expected, stimulation with LPS or CpG did not induce proliferation in *Myd88*^−/−^ and *Myd88*^−/−^ CD19-hBtk B cell fractions (Figure 6E).

Although we did not detect any effects of MyD88-deficiency on αIgM-induced B cell proliferation and survival or expression of activation markers *in vitro*, it cannot be excluded that in an *in vivo* environment MyD88-deficiency may hamper or augment BCR-dependent survival or proliferation signals. Therefore, we investigated whether MyD88-deficiency would influence leukemia development in our *IgH.TE*μ CLL mouse model, in which we previously showed that (i) CLL development is critically dependent on Btk and is accelerated in the presence of the CD19-hBtk transgene, and that (ii) malignant CLL B cells harbor high phosphorylation of Btk, Akt, and S6 (34, 36). To this end, we crossed *IgH.TE*μ mice on the *Myd88*^−/−^ background. Monitoring for the presence of increased frequencies of malignant CD5^+^ B cells in peripheral blood (see Materials and Methods) revealed that *IgH.TE*μ and *Myd88*^−/−^ *IgH.TE*μ mice had a comparable incidence of leukemic disease (Figure S3A). In addition, analysis of the CLL cells for phosphatidylcholine (PtC)-specificity of the BCR, indicative for a B-1 cell origin (37), showed that MyD88-deficiency had no major effect on the usage of the PtC-specific stereotypic BCR (Figure S3B).

From these *in vitro* and *in vivo* experiments, we conclude that the BCR responsiveness of CD19-hBtk B cells is increased, irrespective of the presence of MyD88.

### MyD88 Is Indispensable for Btk-Mediated Autoimmune Disease

**Figure 7:**
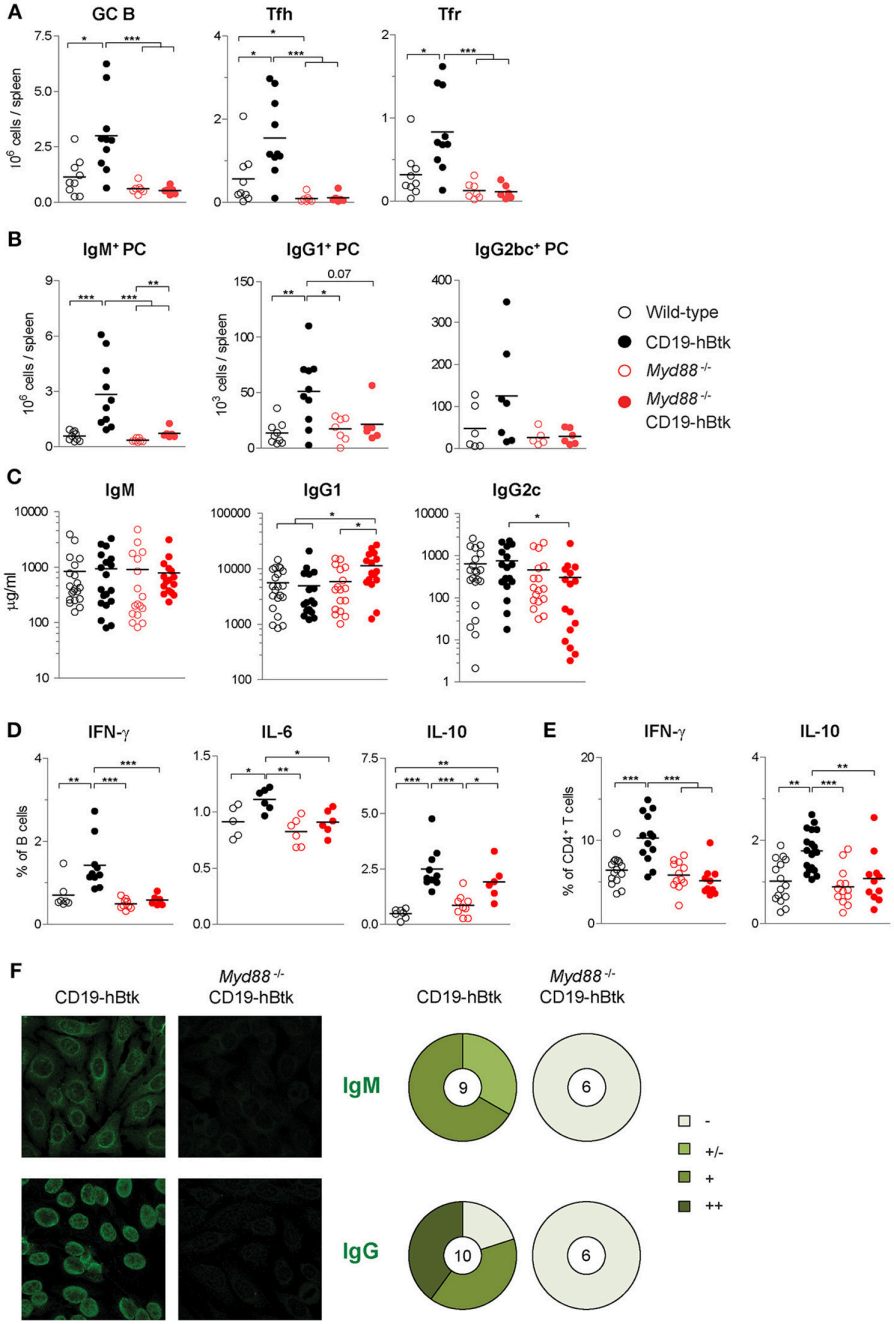
MyD88 is required for Btk-mediated autoimmune disease. **(A)** Absolute numbers of splenic germinal center B cells (GC; CD19^+^IgD^−^CD95^+^), Fol T helper cells (Tfh; CD3^+^CD4^+^CXCR5^+^PD1^+^ FoxP3^−^) and Fol T regulatory cells (Tfr; CD3^+^CD4^+^CXCR5^+^PD1^+^ FoxP3^+^). **(B)** Absolute numbers of splenic IgM^+^, IgG1^+^, and IgG2bc^+^ plasma cells (PC; CD11b^−^IgG1^−^IgGbc^−^IgM^+^CD138^+^, CD11b^−^IgG1^+^CD138^+^, and CD11b^−^IgG2bc^+^CD138^+^, respectively). **(C)** Serum concentrations of IgM, IgG1, and IgG2c, as determined by ELISA. **(D,E)** IFNγ, IL-6, and IL-10 expression in gated B cell fractions **(D)** and CD3^+^CD4^+^ T cell fractions **(E)** upon *in vitro* stimulation with PMA/ionomycin for 4 h in the presence of monensin. Symbols represent individual mice and bars indicate mean values. Graphs represent two to three individual experiments. **(F)** Representative pictures of serum IgM (upper panel) and IgG (lower panel) reactivity with HEp-2 cells and quantification of autoreactivity. Total number of mice analyzed are indicated within the pie charts; –, no staining; +/–, mild staining; +, moderate staining; ++, strong staining. CD19-hBtk and WT mice were 28–33 weeks old; ^*^p < 0.05, ^**^p < 0.01, ^***^p < 0.001 by Mann–Whitney *U*-test.

Investigating autoimmune parameters in aged mice revealed that CD19-hBtk mice had increased numbers of splenic GC B cells, Fol helper T (Tfh) cells, and Fol regulatory T (Tfr) cells compared to WT littermates (Figure 7A; gating strategy in Figures S2A,C,D), as previously observed (8, 16). This increase was fully dependent on MyD88 expression, as *Myd88*^−/−^ CD19-hBtk mice had splenic GC B cell, Tfh and Tfr numbers similar to WT or *Myd88*^−/−^ mice (Figure 7A). In addition, the increase of IgM^+^, IgG1^+^, and less prominently, IgG2bc^+^ plasma cells in the spleens of CD19-hBtk mice was MyD88-dependent (Figure 7B; gating strategy in Figures S2A,E). IgM^+^ plasma cells in the BM were also MyD88-dependently increased (Figure S4A). IgG1^+^ plasma cells in BM were increased in MyD88-deficient mice, as was previously described, and increased further in Btk-overexpressing MyD88-deficient mice (Figure S4A). Total serum IgM levels were not different between the four groups of mice (Figure 7C). Likewise, the increased numbers of IgG1^+^ plasma cells in the spleens of CD19-hBtk mice was not reflected by increased total IgG1 concentrations in the serum, compared with the three other groups of mice (Figures 7B,C). We observed increased total IgG1 and decreased IgG2c levels in the serum of *Myd88*^−/−^ CD19-hBtk mice, when compared with CD19-hBtk mice, which reflected the numbers of plasma cells in BM rather than spleen (Figures 7B,C; Figure S4A).

Next, we investigated the cytokine producing capacity of splenic B and T cells. We observed that the increase of the proportions of IL-6^+^ and IFNγ^+^ cells in the splenic B cell populations of CD19-hBtk mice was MyD88-dependent (Figure 7D). In contrast, the increase in IL-10^+^ B cells in CD19-hBtk mice appears to be MyD88-independent (Figure 7D). A separate analysis of Fol and MZ B cells showed that the profiles for IFNγ^+^, IL-6, and IL-10 across the four groups of mice were similar in the two B cell subsets (not shown). For CD5^+^ B-1 we observed that the production of the three cytokines was slightly reduced in CD19-hBtk mice, in line with our findings described above (Figures 4, 5), as well as in *Myd88*^−/−^ CD19-hBtk mice (not shown). The observed increase of cytokine production, including IFNγ^+^ and IL-10, by splenic T cells from in CD19-hBtk mice was not observed in T cells from *Myd88*^−/−^ CD19-hBtk mice (Figure 7E).

Analysis of autoimmune pathology by immunohistochemistry showed numerous perivascular B and T lymphocyte infiltrates in the salivary glands of CD19-hBtk mice, which were completely absent in *Myd88*^−/−^ CD19-hBtk mice (Figure S4B), as well as in WT and *Myd88*^−/−^ mice (data not shown). MyD88 was also required for IgM^+^ or IgG2c^+^ glomerular immune complex depositions in the kidneys, which were present in CD19-hBtk but hardly in *Myd88*^−/−^ CD19-hBtk mice (Figure S4C). The production of IgM autoantibodies (mainly cytoplasmic) and anti-nuclear IgG autoantibodies, as seen in CD19-hBtk mice, was absent in *Myd88*^−/−^ CD19-hBtk mice (Figure 7F). It is of note that the anti-nuclear auto-antibodies in CD19-hBtk mice were reactive to dsDNA and chromatin (8, 16), RNA polymerase RNP-A, RNP-C, and Smith antigen SmB (Figure S4D; Table S2), representing auto-antibody specificities that were shown to be dictated by TLR7 and TLR9 (17).

Taken together, these data show that MyD88 is required for the development of all hallmarks of autoimmune pathology in CD19-hBtk mice with increased BCR responsiveness.

## Discussion

Btk is a critical kinase in the BCR signaling pathway and is known to interact with various proteins that are downstream of TLRs. The interplay between the BCR and TLR signaling is thought to be crucial for the pathogenesis of autoimmune disease. Our findings provide evidence that TLR signaling is critical in systemic autoimmunity driven by overexpression of Btk in transgenic mice, which is characterized by the induction of TLR7/9-associated auto-antibody specificities, including dsDNA, histone, RNP-A, RNP-C, and SmB.

Analysis of the functional consequences of BCR and TLR stimulation in our CD19-hBtk mouse model revealed that substantial complexity exists regarding the interplay of the two signaling pathways. Btk overexpression amplified S6 signaling in the context of IgM stimulation, but no effects were observed on signaling levels upon TLR stimulation, even though we detected decreased protein levels of TLR9 in CD19-hBtk B cells compared to WT controls. In parallel, we found that Btk overexpression increased survival and proliferation of B cells when these were stimulated through BCR engagement, but reduced proliferation after CpG or CpG/αIgM stimulation. Nevertheless, using different functional readouts, clear additive, or synergistic effects were observed. These include upregulation of surface markers such as CD25, CD80, and CD86 and the capacity of CD19-hBtk B cells to produce significantly increased IL-10 levels after CpG stimulation, as compared to WT B cells. Nevertheless, enhanced IL-10 expression was only seen in Fol and MZ B cells and not in CD5^+^ B-1 cells. Along these lines, we found that in aging CD19-hBtk mice Fol B cells and CD5^+^ B-1 cells expressed increased levels of IL-6 and IFNγ, but this was essentially not increased upon *in vitro* stimulation with PMA/ionomycin, αIgM, or CpG. We also noticed that TLR9 engagement by CpG increased the capacity of MZ B cells to produce IL-6 to levels that were beyond those reached by PMA/ionomycin stimulation. In contrast, CpG stimulation of MZ B cells appeared to decrease their capacity to produce IFNγ. Despite the observed complexity of TCR and BCR interplay exposing both synergistic and opposite outcomes, we established that MyD88-deficient CD19-hBtk transgenic mice did not develop autoimmune symptoms. Therefore, we conclude that TLR signaling is crucial for the induction of Btk-driven autoimmune disease.

Various molecular mechanisms may connect Btk to both BCR and TLR signaling. First, Btk is phosphorylated downstream of the BCR and can directly interact with several components of the TLR signaling pathway. These include the Toll/IL-1R homology (TIR) domain, which is the intracellular signaling module of TLR, the adapters MyD88 and MyD88 adapter-like (MAL) and IL-1R-associated kinase-1 (IRAK-1) (9, 27). In particular, Kenny et al. showed that Btk is essential for co-localization of the BCR and TLR9 within an auto-phagosome-like compartment (30). The authors found synergistic upregulation of activation markers, which is in concordance with our findings. However, we did not observe a kinase-independent role for Btk in synergistic IL-6 production in response to CpG and αIgM in splenic B cells. Second, upon BCR engagement Btk phosphorylates the B-cell adaptor for phosphoinositide 3-kinase (BCAP), which provides a binding site for PI3K (38). Interestingly, BCAP also links TLR signaling to PI3K activation (39, 40) and contains a TIR domain, which is used by TLR signaling adapters including MyD88. As a result, it is conceivable that BCAP reduces the availability of MyD88 for activation of NF-κB. Third, PLCγ2, which is a direct substrate of Btk, can interact with another TIR-domain containing adapter molecule, the B cell adaptor protein with ankyrin repeats (BANK1) (41) which shows genetic association with SLE in GWAS (42). BANK1 augments TLR7/TLR9 signaling and was reported to control CpG-induced IL-6 secretion (43). B cell IL-6 production, which can be enhanced by IFNγ, promotes Tfh cell differentiation and initiates spontaneous GC formation (44). Therefore, it is likely that increased IL-6 production by Btk-overexpressing B cells is a major driver of autoimmunity in CD19-hBTK mice. In our crosses with MyD88-deficient mice we could show that enhanced expression of both IL-6 and IFNγ in CD19-hBtk mice is TLR-dependent.

IL-10-producing B cells are important in autoimmune diseases, as IL-10 is a proliferation factor for B cells and has well-known regulatory functions (45–47). We observed that in PMA/ionomycin stimulation experiments IL-10 production by CD19-hBtk B cells was independent of MyD88 (Figure 7D) and independent of CD40L expression (16). However, stimulation with TLR ligands strongly induced IL-10-producing CD19-hBtk B cells, but WT B cells showed low responsiveness. This is in line with the previously reported finding that Btk is required for TLR-induced IL-10 production by B cells (48, 49) and that B cells from lupus-prone mice upregulate IL-10 production in response to TLR stimulation, but not to BCR or CD40 engagement (50). Furthermore, we found that CD19-hBtk B cells specifically increased IL-10 production upon combined BCR-TLR stimulation over TLR stimulation alone, whereas this remained unchanged in WT B cells. This finding demonstrates that regarding IL-10 production autoimmune CD19-hBtk B cells are very sensitive to dual ligation, which is in stark contrast with the limited responsiveness of WT B cells.

We also found that all splenic B cell subsets produced IL-10. Peritoneal CD5^+^ B-1 cells were previously shown to rely on Btk for the production of IL-10, as B cells with low Btk expression had decreased levels of IL-10 mRNA, compared with B cells with physiological Btk levels (51). Although the number of CD5^+^ B-1 cells is significantly increased in CD19-hBtk mice, we did not observe increased proportions of IL-10^+^ cells within the splenic CD5^+^ B-1 population in CD19-hBtk mice, compared with WT. It was recently reported that Btk is not required for the maintenance of the B-1 cell pool (52). Therefore, it is very well-possible that Btk overexpression mainly affects the generation of CD5^+^ B-1 cells during development and not the IL-10 production by CD5^+^ B-1 cells in adult mice. The increase in IL-10 production and the synergistic responsiveness of CD19-hBtk Fol and MZ B cells might point to an attempt of B cells to control the autoimmune response, potentially via TLR9 signaling (17, 23, 53). However, elevated IL-10 serum levels were found in SLE patients with active disease (54), suggesting that IL-10-producing autoimmune B cells could be disease-promoting, e.g., by the effects of IL-10 on B cell survival (45).

The increase in IFNγ^+^ and IL-6^+^ CD19-hBtk B cells upon PMA/ionomycin stimulation was fully dependent on MyD88 expression and *in vitro* Btk inhibition for several hours did not reduce IFNγ and IL-6 to WT levels. This suggests that *in vivo* TLR-mediated B cell activation is crucial for the increase in IFNγ^+^ and IL-6^+^ CD19-hBtk B cells. Further experiments are required to identify the molecular mechanisms that are responsible for the altered cytokine production capacity upon long-lasting *in vivo* B cell activation, apparently resulting in rewiring of signal transduction pathways and turning these B cells refractory to Btk inhibition.

We demonstrated that TLR signaling is important in hBtk-mediated autoimmune disease, although the *in vitro* BCR responsiveness of CD19-hBtk B cells, including downstream signaling, survival and proliferation, was enhanced in a MyD88-independent fashion. However, spontaneous GC formation, increased plasma cell differentiation, induction of Tfh cells, increased production of IFNγ and IL-6, and the presence of ANAs in serum, were clearly abrogated in the absence of MyD88. It was recently reported that circulating chromatin in apoptotic cell micro-particles induced SLE, which was dependent on MyD88 expression (24). This suggests that autoreactive BCRs bind chromatin in apoptotic material, providing a strong survival signal by synergistic signaling with nucleic acid-sensing TLRs. In addition, BCR-TLR synergism can drive AID expression and thereby promote class-switching in T cell independent responses (55). TLR signaling was also shown to be required for amplification of GC responses, whereby B-cell-intrinsic TLR responsiveness was upregulated during GC reactions (56), implicating TLR signaling also in later stages of B cell activation and differentiation.

Our findings demonstrate that enhanced BCR signaling in CD19-hBtk B cells on its own is not sufficient for the development of autoreactive responses. TLR expression in cell types other than B cells could well-contribute to Btk-driven autoimmunity, particularly because TLR signaling in dendritic cells (DCs) and lymph node stromal cells is relevant for activation of (autoreactive) B cells (22, 57, 58). Thus, the activation of autoreactive B cells relies on TLR signaling in B cells and potentially also in other cells, such as DCs and lymph node stromal cells.

In summary, we have shown that TLR-mediated activation is important for Btk-driven autoimmune disease, partly by synergistically enhancing signaling responses of autoreactive B cells. Next to mouse studies concerning Btk inhibition in autoimmune diseases, currently clinical trials are ongoing with several Btk inhibitors in autoimmune disease patients, including RA (CC-292, HM71224, M2951, and GS-4059) and SLE (BIIB068, MSC2364447C, and M2951) (www.clinicaltrials.gov). Based on our current findings, together with the observed increase in BTK protein expression levels in circulating B cells from patients with RA and SjS (15), it is attractive to speculate that Btk inhibition will dampen both BCR pathway activation and synergistic BCR/TLR signaling in patients with systemic autoimmune disease.

## Author Contributions

JR designed the research studies, performed experiments, analyzed the data, and wrote the manuscript. MdB, SP, and MA performed experiments and analyzed the data. OC and RH contributed to the research design and the writing of the manuscript and supervised the study. All co-authors approved the final manuscript.

### Conflict of Interest Statement

The authors declare that the research was conducted in the absence of any commercial or financial relationships that could be construed as a potential conflict of interest.
