# Supplementary material for: Toll-Like Receptor Signaling Drives Btk-Mediated Autoimmune Disease
Source: Front Immunol. 2019 Jan 30;10:95. doi: 10.3389/fimmu.2019.00095 (PMC6363707; doi:10.3389/fimmu.2019.00095)
Supplement: Supplementary file 1 [file Data_Sheet_1.pdf]

SUPPLEMENTAL MATERIAL TO:

Toll-like receptor signaling drives Btk-mediated autoimmune disease

Jasper Rip[1], Marjolein J.W. de Bruijn[1], Marjolein K. Appelman[1], Simar Pal Singh[1,2], Rudi W. Hendriks[1,#] and Odilia B.J. Corneth[1,#]

[1] Department of Pulmonary Medicine, Erasmus MC Rotterdam, The Netherlands
[2] Department of Immunology, Erasmus MC Rotterdam, The Netherlands
[#] Authors contributed equally

Table S1A. Antibodies used for FACS

Antigen	Fluorochrome	Clone	Manufacturer
Flow cytometry			
B220	AF700	RA3-6B2	BD Biosciences
CD1d	BV421	1B1	BD Biosciences
CD3	APC-efl780	17A2	eBioscience
	BV421	145-2C11	BD Biosciences
CD4	AF700	GK1.5	eBioscience
	PCP-cy5.5	RM3-5	eBioscience
CD5	APC	Ly-1	eBioscience
CD11b	AF700	M1/70	eBioscience
CD19	APC-ef780	1D3	BD Biosciences
	PCP-Cy5.5	eBio1D3	eBioscience
CD21/35	FITC	eBio4E3	eBioscience
	BV605	7G6	BD Biosciences
CD23	PE-Cy7	B3B4	BD Biosciences
CD25	BV605	PC61	Biolegend
CD43	PE	S7	BD Biosciences
CD80	PCP-cy5.5	GL1	BD Biosciences
CD86	PE-cy7	GL1	BD Biosciences
CD95	PE-CF594	Jo2	BD Biosciences
CD138	APC	281-2	BD Biosciences
IgD	FITC	11-26c.2a	eBioscience
	BV711	11-26c.2a	BD Biosciences
IgM	PE-Cy7	II/41	BD Biosciences
IgG1	FITC	A85-1	BD Biosciences
CTLA4	PE	UC10-4F10-11	BD Biosciences
CXCR5	Biotin	2G8	BD Biosciences
FoxP3	FITC	FJK-16s	eBioscience
ICOS	APC	C398.4A	eBioscience
IFN-γ	PE-Cy7	XMG1.2	eBioscience
IL-6	PE	MP5-20F3	BD Biosciences
IL-10	FITC	JES5-16E3	eBioscience
PD-1	BV421	J43	BD Biosciences
TLR7	PE	A94B10	BD Biosciences
TLR9	PE	J15A7	BD Biosciences
Streptavidin	APC-efl780	-	eBioscience
	PE-Cy7	-	eBioscience
	BV711	-	BD Biosciences

Table S1B. Antibodies used for Phosphoflow

Antigen	Fluorochrome	Clone	Manufacturer
Phosphoflow			
CD3	BV421	145-2C11	BD Bioscience
B220	AF700	RA3-6B2	BD Biosciences
IgD	BV711	11-26c.2a	BD Biosciences
IgM	PE-Cy7	II/41	BD Biosciences
pCD79a (Y182)	AF647	D1B9	Cell signaling Technologies
pSyk (Y348)	PE	L120-722	BD Biosciences
pAkt (T308)	-	D25E6	Cell signaling Technologies
pAkt (S473)	-	D9E	Cell signaling Technologies
pPLCγ2 (Y759)	AF647	K86-689.37	BD Biosciences
pS6 (S240/S244)	-	D68F8	Cell signaling Technologies
anti-Rabbit	PE	-	Jackson ImmunoResearch
Streptavidin	BV786	-	BD Biosciences

Table S1C. Antibodies used for MACS procedures

Antigen	Fluorochrome	Clone	Manufacturer
CD5	Biotin	53-7.3	BD Bioscience
CD11b	Biotin	M1/70	eBioscience
CD138	Biotin	281-2	BD Bioscience
CD43	Biotin	eBioR2/60	eBioscience
CD95	Biotin	Jo2	BD Bioscience
Gr-1	Biotin	RB6-8C5	eBioscience
TER-119	Biotin	TER119	eBioscience

Table S2. Autoantibody reactivity in aged WT, CD19-hBtk mice and *Myd88*$^{-/-}$ CD19-hBtk mice.

Genotype		Reactivity of autoantibodies*)
**Wild-type**	#1	-
	#2	-
	#3	-
**CD19-hBtk**	#1	Histones (++), RNP-A (++), RNP-C (++)
	#2	RNP-A (++), RNP-C (+)
	#3	Histones (++), RNP-A (++), RNP-C (++), SmB (+)
	#4	RNP-A (+)
	#5	Histones (++), RNP-A (++), RNP-C (++)
	#6	Histones (++), RNP-A (++), RNP-C (++)
	#7	Histones (++), RNP-A (+), RNP-C (+)
Myd88$^{-/-}$ **CD19-hBtk**	#1	-
	#2	-
	#3	-

*) Autoantibody reactivity in 28-33 week-old mice, as measured by Line Immunoblot Assay.

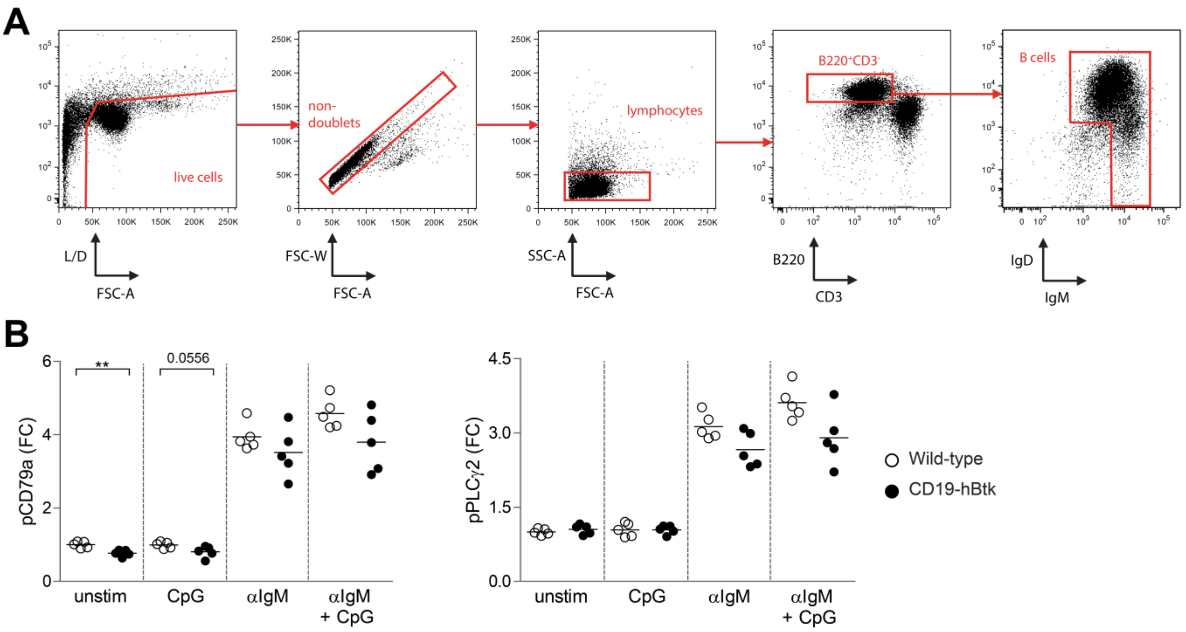

Supplementary Figure 1. Gating strategy phosphoflow and analysis of pCD79a and pPLCγ2 in B cells after stimulation.

(**A**) Gating strategy for live cells by viability marker staining, followed by gating for single lymphocytes. From live lymphocytes, we gated for splenic B cells ($B220^{+}CD3^{-}IgD^{+}IgM^{+}$). (**B**) Fold Change (FC) increase of median fluoresence intensity (MFI) values compared to WT unstimulated (which was set to 1.0) for phosphorylation of CD79a and PLCγ2 in B cells that were activated by the indicated stimuli. Symbols represent individual mice and bars indicate mean values.CD19-hBtk and WT mice were 8-10 weeks old; n = 4-5 per group; $^{**}p<0.01$ by Mann-Whitney U test.

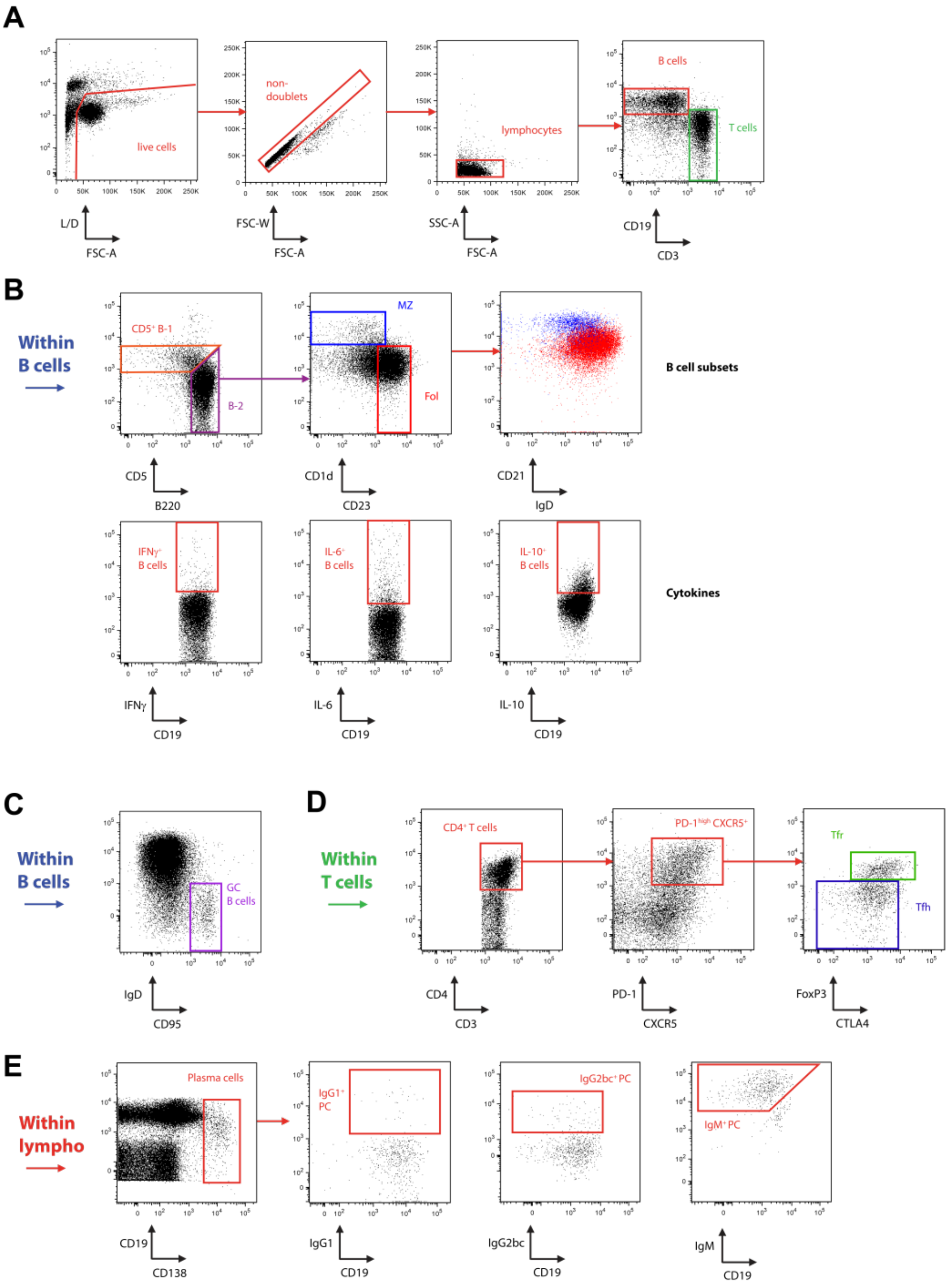

Supplementary Figure 2. Gating strategy for cytokine stainings, B cell subsets and plasma cells. (**A**) For all stainings, we first gated for single live B and T cells, based on CD19 and CD3 expression. (**B**) For B cell subset cytokine staining, we gated within CD19^{+}CD3^{-} B cells for CD5^{+} B-1 cells (CD19^{+}B220^{int}CD5^{+}), marginal zone B cells (CD19^{+}B220^{+}CD1d^{high}CD21^{high}CD23^{-}IgD^{-}) and follicular B cells (CD19^{+}B220^{+}CD1d^{-}CD21^{+}CD23^{+}IgD^{+}). Expression of IFNγ, IL-6 and IL-10 within total B cells are shown as examples. (**C**) From B cells, we gated for germinal center B cells (CD19^{+}IgD^{-}CD95^{+}). (**D**) From T cells, we gated on CD4+ cells that highly express PD-1 and are CXCR5-positive. Foxp3 and CTLA4 were used to separate T follicular helper cells (Tfh; CD3^{+}CD4^{+}CXCR5^{+}PD1^{+}FoxP3^{-}) and T follicular regulatory cells (Tfr; CD3^{+}CD4^{+}CXCR5^{+}PD1^{+}FoxP3^{+}); representative flow cytometry graphs for these gating strategies. (**E**) From CD11b-negative lymphocytes, we gated for IgM^{+}, IgG1^{+} and IgG2bc^{+} plasma cells (PC; CD11b^{-}IgG1^{-}IgG2bc^{-}IgM^{+}CD138^{+}, CD11b^{-}IgG1^{+}CD138^{+} and CD11b^{-}IgG2bc^{+}CD138^{+} respectively).

A

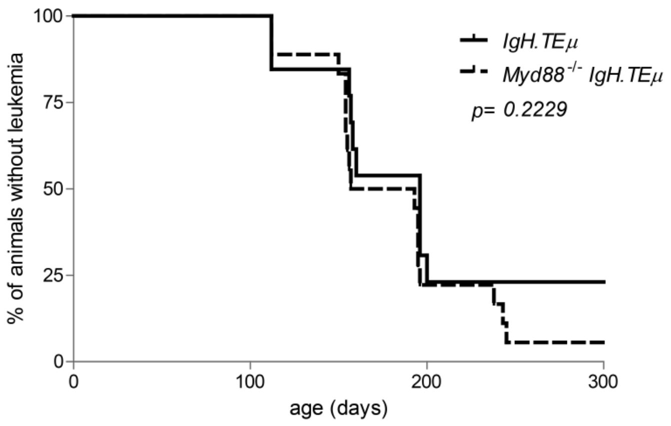

B

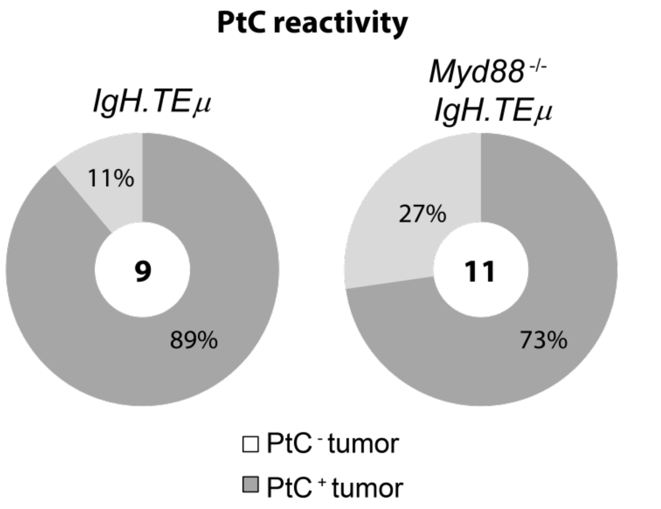

Supplementary Figure 3 (**A**) Retrospective Kaplan-Meier incidence curve showing *IgH.TEμ* mice (solid line) and *Myd88* $^{-/-}$ *IgH.TEμ* mice (dotted line). (**B**) Pie chart summarizing frequencies of splenic PtC-recognizing (PtC^{+}) and non PtC-recognizing (PtC^{-}) CLL cases from nine *IgH.TEμ* mice (*left*) and eleven *Myd88* $^{-/-}$ *IgH.TEμ* mice (*right*), as indicated. Log Rank test was performed to calculate the significance for survival differences between indicated groups.

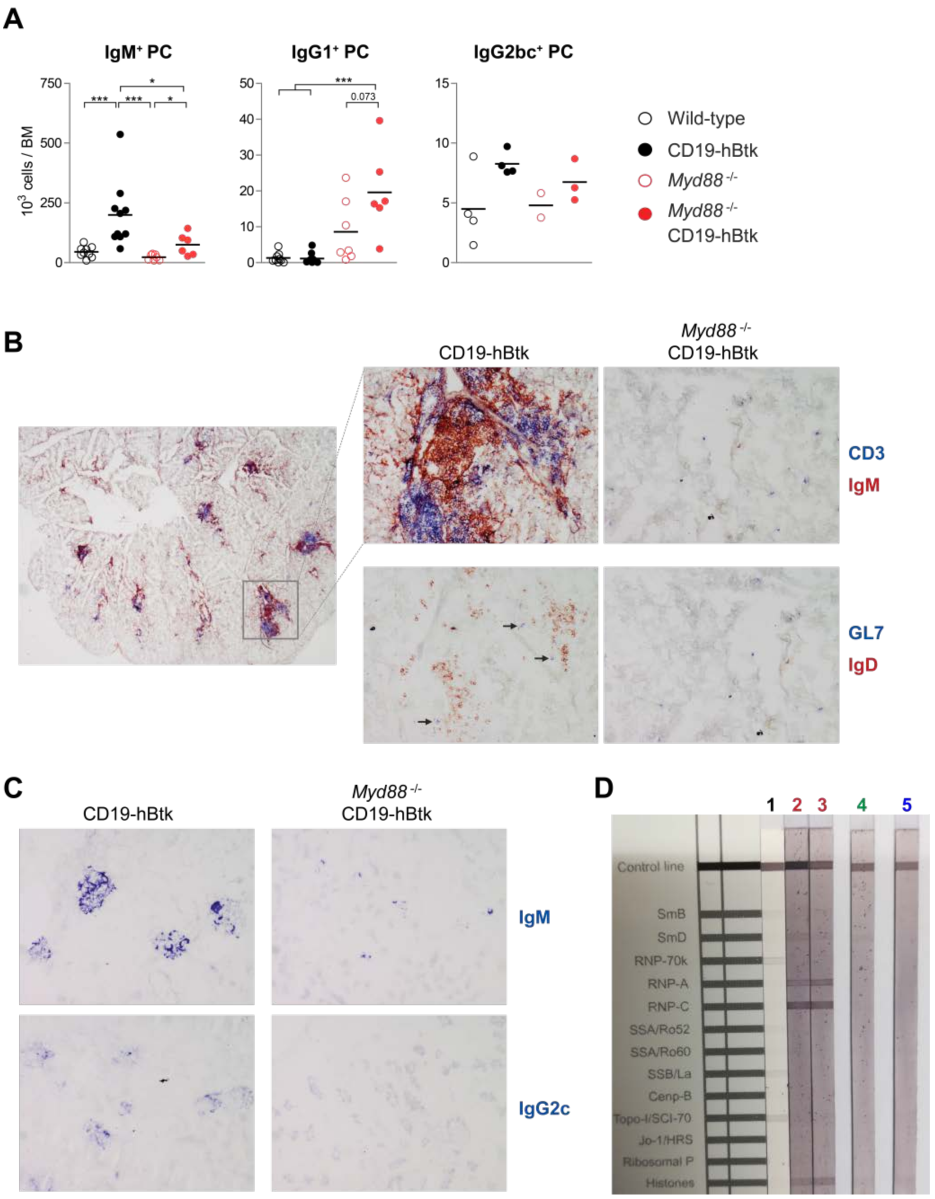

Supplementary Figure 4. The autoimmune phenotype of CD19-hBTK mice, compared with *Myd88*$^{-/-}$ CD19-hBtk mice.

(**A**) Absolute numbers of bone marrow (BM) IgM^{+}, IgG1^{+} and IgG2bc^{+} plasma cells (PC; CD11b^{-}IgG1^{-}IgGbc^{-}IgM^{+}CD138^{+}, CD11b^{-}IgG1^{+}CD138^{+} and CD11b^{-}IgG2bc^{+}CD138^{+} respectively). Numbers are per single femur. (**B**) Histological analysis of salivary gland sections for CD3^{+} T cells (blue) and IgM^{+} B cells (red) (top panel) and GL7^{+} GC B cells (blue) and IgD^{+} B cells (red) (lower panel) at 28-33 weeks of age. Representative pictures are shown, n= 3 per group. (**C**) Histological analysis for IgM (upper panel) and IgG2c (lower panel) immune complex deposition in kidney glomeruli at 28-33 weeks of age. Representative pictures are shown, n= 3 per group. (**D**) Line Immunoblot Assay (LIA) for extractable nuclear antigens. The indicator reference is located on the left. On top of the LIA strips, five immunoblots are indicated by numbers: the cut-off control (1) and representative pictures of CD19-hBtk (2,3), wild-type (4) and *Myd88*$^{-/-}$ CD19-hBtk (5) mice. Only CD19-hBtk mice stained positive for autoantibodies, in these specific cases for RNP-A, RNP-C and histones.
